# Enhanced access to anthropogenic food waste is related to hyperglycemia in raccoons (*Procyon lotor*)

**DOI:** 10.1093/conphys/coy026

**Published:** 2018-06-13

**Authors:** Albrecht I Schulte-Hostedde, Zvia Mazal, Claire M Jardine, Jeffrey Gagnon

**Affiliations:** 1 Department of Biology, Laurentian University, 935 Ramsey Lake Rd., Sudbury, ON, Canada; 2 Centre for Evolutionary Ecology and Ethical Conservation (CEEEC), Laurentian University, Sudbury, ON, Canada; 3 Department of Pathobiology, Canadian Wildlife Health Cooperative, Ontario Veterinary College, University of Guelph, 50 Stone Rd. E, Guelph, ON, Canada

**Keywords:** anthropogenic food source, glucose metabolism, mesocarnivore, urban ecology

## Abstract

Urban landscapes have well-known effects on wildlife populations. Many species of urban wildlife feed on anthropogenic food wastes, and little is known regarding the sub-lethal physiological consequences of this novel diet. We use samples from three populations of raccoons to test the hypothesis that access to anthropogenic food waste will lead to elevated body mass, blood glucose and serum leptin. Each population varied in their presumed access to food waste. We found that raccoons from the site with the highest presumed access to food waste were significantly heavier and had significantly higher levels of glycated serum protein (GSP, a marker of elevated blood glucose). In addition, GSP concentration was positively related to body mass. No significant differences in serum leptin were detected, nor was serum leptin related to body mass. Urban diets may have significant physiological consequences for urban wildlife related to glucose metabolism. Further research will be needed to determine the evolutionary consequences of the novel urban diet, and whether adaptation is occurring.

## Teaser

Cities are expanding, and urban wildlife, including raccoons, are adapting by feeding on food waste. We found that raccoons with greater access to food waste were heavier and had elevated blood glucose levels, and suggest further research to examine the physiological consequences of urban living in wildlife.

## Introduction

Cities and their associated infrastructure are rapidly expanding as human populations become increasingly urbanized (Grimm *et al.*, 2008). These urban landscapes are highly modified from their natural state, and offer novel habitats for wildlife (Shochat *et al.*, 2006). Urban processes may differ dramatically from natural processes that have governed the ecology and evolution of species in natural habitats (Shochat *et al.*, 2006). While it is well-known that species distributions may vary based on the degree of urbanization of the landscape, less well understood are the sub-lethal consequences of urbanization on wildlife (Birnie-Gauvin *et al.*, 2016).

The consequences of human-induced environmental change, including urbanization, on nutritional physiology and ecology of wildlife are understudied and require further research (Birnie-Gauvin *et al.*, 2017). Predictable anthropogenic food subsidies can have important effects on wildlife populations (Oro *et al.*, 2013). In cities, anthropogenic food subsidies, particularly human refuse, may have consequences for urban wildlife (Bateman and Fleming, 2012) including altering predator–prey interactions (Rodewald *et al.*, 2011) and spatial distributions (Prange *et al.*, 2004; Bozek at al., 2007). Medium-sized carnivores, in particular, may thrive in urban habitats, in part, because of access to anthropogenic food waste (Bateman and Fleming, 2012). Population densities may increase due to higher litter sizes resulting from the enhanced food resources associated with anthropogenic food waste (Prange *et al.*, 2003).

Anthropogenic food waste is different from a natural diet for wildlife (Bateman and Fleming, 2012; Murray *et al.* 2015). The consumption of anthropogenic food waste should have physiological consequences for wildlife, in particular related to glucose and fat metabolism, but few studies have examined this issue at the population level. Banks *et al.* (2003) found that a subset of yellow baboons (*Papio cynocephalus*) with access to anthropogenic food waste had higher body mass with corresponding insulin resistance and elevated serum levels of glucose. These yellow baboons also had higher levels of leptin (Banks *et al.*, 2003), a peptide hormone produced primarily within adipocytes (Friedman and Halaas, 1998). Leptin is linked to appetite control, fat metabolism and energy balance in many mammals (Friedman and Halaas, 1998), and is associated with adiposity in several species (Suzuki *et al.*, 2004; Spady *et al.*, 2009).

The raccoon (*Procyon lotor*) is a mesocarnivore from the family Procyonidae, and is a notorious invasive species and inhabitant of cities (e.g. Okabe and Agetsuma, 2007; Garcia *et al.*, 2012) with a broad and omnivorous diet (Lotze and Anderson, 1979). While there is evidence of ecological effects of anthropogenic food waste on raccoon populations (e.g. Prange *et al.*, 2003; 2004), there is also further evidence related to individual health. For example, the presence of dental caries and poor periodontal health of adult raccoons was highest in raccoons with access to anthropogenic food waste at a campground compared to raccoons from a rural area (Hungerford *et al.*, 1999). Glucose metabolism associated with large body mass (‘obesity’) has also been investigated, with a 10.5-kg raccoon from an animal sanctuary being diagnosed with diabetes and successfully treated with insulin and dietary management (McCain *et al.*, 2008).

If glucose metabolism, adiposity, and body weight are affected by access to, and consumption of, anthropogenic food waste then we predict that raccoons with greater access to such food sources will exhibit higher body mass, higher serum leptin levels and evidence of hyperglycemia (elevated blood sugar) compared with raccoons with reduced access to anthropogenic food waste. We used an existing bank of serum from raccoons sampled from sites that varied in the presumed access to anthropogenic food waste by raccoons to test our predictions.

## Methods

### Serum samples

Procedures for trapping and handling raccoons were approved by the Animal Care Committee at the University of Guelph following the guidelines of the Canadian Committee on Animal Care and have been described previously (Jardine *et al.*, 2011; Bondo *et al.*, 2016). Briefly, raccoons were trapped in each area using Tomahawk live traps (sizes 106 and 108; Tomahawk Live Trap Co. Tomahawk, Wisconsin, USA) baited with sardines or cat food. Raccoons were anaesthetized using an intramuscular injection of 0.025 mg/kg dexmedetomidine hydrochloride (Dexdomitor 0.5 mg/ml; Pfizer Animal Health, Kirkland, Quebec, Canada) and 5 mg/kg ketamine hydrochloride (Vetalar 100 mg/ml; Bioniche Animal Health, Belleville, ON, Canada) before being removed from the trap. Sex, age class (adult or juvenile, based on animal size and teeth wear/staining) and mass were recorded for each animal. Blood (≤ 5ml) was collected from the jugular vein. After samples were collected, raccoons were given an anaesthetic reversal agent, 0.25 mg/kg atipamezole (Antisedan 5 mg/ml; Pfizer Animal Health, Kirkland, Quebec, Canada) and placed back into traps to recover from the anaesthetic before being released at the location of capture. Blood samples were kept cool during transport back to the laboratory and serum was extracted within 12 h and stored frozen at −70°C prior to being shipped to Laurentian University for analysis.

Raccoon samples were collected from 3 different location types in southern Ontario. Each location type had a varying degree of access to anthropogenic food waste. Samples were collected from the ‘high access’ to anthropogenic food waste site located on the grounds Toronto Zoo (Scarborough, Ontario), in 2007. This site provided daily access to anthropogenic food waste from garbage bins and dumpsters around the property, including anthropogenic food waste from on-site restaurants and similar sites. Samples were collected from the ‘moderate access’ sites, which included three conservation areas in the Grand River Watershed, Ontario, in 2012. These areas are forested green spaces in urban areas that are used for recreational activities (e.g. hiking, biking, dog walking, bird watching and picnics). People do not live in these sites, but the sites are located in residential areas (so people live in close proximity to these sites). These sites provided access to anthropogenic food waste through garbage bins accessible during weekly municipal garbage collection. Samples were collected from the ‘low access’ to anthropogenic food waste site in a farming area in the Grand River Watershed, Ontario, in 2012. This site had less access to anthropogenic food waste in part because of the relatively limited number of people present on the property. We restricted analysis of the raccoon samples to adult animals sampled during the months of July and August to account for seasonal variations in body weight and glycaemia.

### Glycated serum protein assay

A commercial rat glycated serum protein (GSP) assay was used to quantify the GSP concentrations in each raccoon serum sample (Crystal Chem, IL). This assay detects the degree of protein glycation in biological samples and provides a 2–3 day window of circulating glucose. All samples were assayed in duplicate as per the manufacturer’s guidelines.

### Leptin assay

A commercial rat leptin enzyme immunosorbent assay was used to quantify serum leptin concentration (crystal Chem, IL). The leptin antibody used in this kit has known cross-reactivity with mouse, rat and human leptin. Leptin levels correlate with increased adiposity. Detected serum leptin levels (2–3 ng/ml) were similar to a published report using a canine leptin radioimmuneassay (PMID) 15945076.

### Statistical analysis

Statistical analysis was completed using GraphPad Prism software (La Jolla, CA). Comparisons between the three sites for body weight, GSP and leptin were completed using a one-way ANOVA with a Tukey’s post hoc test to make pairwise comparisons. Comparison between sex and access to anthropogenic food waste on each parameter (GSP and body weight) were completed using a two-way ANOVA. Pearson’s correlation between GSP concentration and body weight were completed for combined data at all sites. We adjusted *α* to 0.05/3 = 0.0167 to correct for three statistical tests related to GSP, leptin and body mass.

## Results

Samples were collected from a total of 60 raccoons, including *n* = 23 (7 females, 16 males) from the ‘high access’ site (the grounds of the Toronto Zoo), *n* = 20 (12 females, 8 males) from the ‘medium access’ site (conservation areas in the Grand River Watershed, Ontario) and 17 raccoons (11 females, 5 males) from the ‘low access’ site (farming area in the Grand River Watershed, Ontario) (see Table 1 for summary of data).

**Table 1:** Summary statistics (mean ± SEM) for raccoons captured in areas with high, moderate and low access to anthropogenic food waste

	High (*n* = 23)	Moderate (*n* = 20)	Low (*n* = 17)
Body weight (kg)	8.40 ± 0.354	6.47 ± 0.334	5.42 ± 0.340
GSP (μM)	102 ± 6.02	51.4 ± 4.51	45.6 ± 5.98
Leptin (ng/ml)	3.39 ± 0.525	3.41 ± 0.507	2.78 ± 0.288

**Figure 1:**
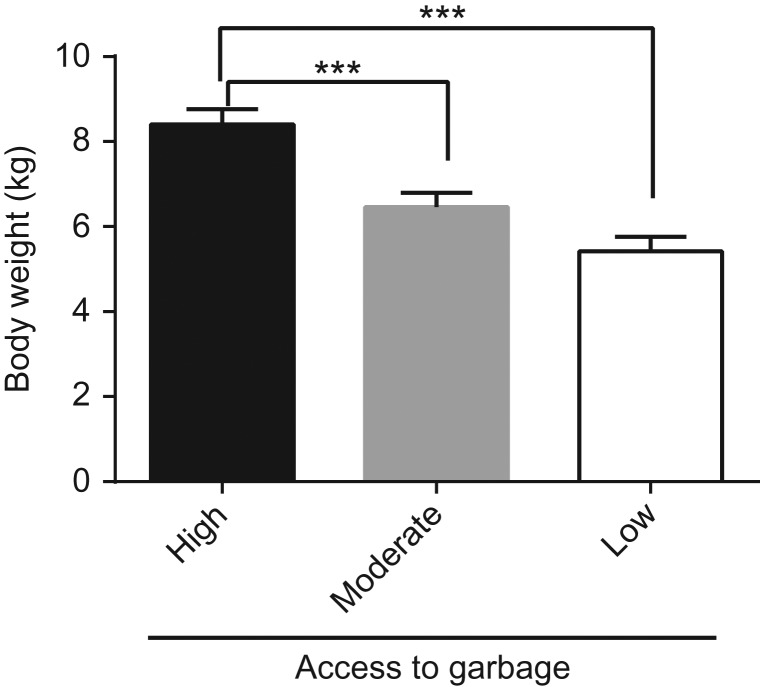
Effect of access to garbage on raccoon body weight. Body weight was measured from raccoons with high, moderate and low access to garbage. Data is presented mean ± SEM in kg. Data was analyzed using a one-way ANOVA and a Tukey’s post hoc test. *n* = 23, *n* = 20 and *n* = 16 for the high, moderate and low access to garbage, respectively. **** = *P* < 0.001 and *** = *P* < 0.001

There was a significant difference (*P* < 0.001, *F* = 19.7, df = 2, 57) in body weight among the three groups of raccoons (Fig. 1). Raccoons with high access to anthropogenic food waste were significantly heavier than those with moderate and low access to anthropogenic food waste (*P* < 0.001). No significant difference (*P* > 0.05) was observed between the mean body weights of the raccoons with moderate and low access to anthropogenic food waste.

**Figure 2:**
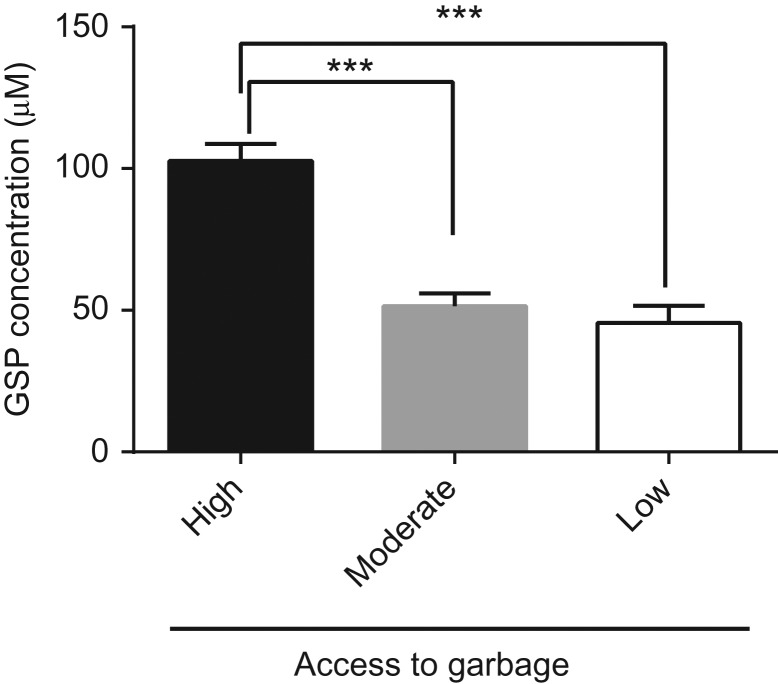
Effect of access to garbage on raccoon GSP concentration. Serum samples were assayed for GSP from raccoons with high, moderate and low access to garbage. Data is presented mean ± SEM in μM. Data was analyzed using a one-way ANOVA and a Tukey’s post hoc test. *n* = 23, *n* = 20 and *n* = 16 for the high, moderate and low access to garbage, respectively. ***P < 0.001

GSP concentration was significant different in among the three groups of raccoons (*P* < 0.001, *F* = 33.13, df = 2, 56—Fig. 2). Raccoons with high access to anthropogenic food waste had significantly higher GSP levels than those with moderate and low access to anthropogenic food waste (*P* < 0.001). No significant difference (*P* > 0.05) was observed between the raccoons with moderate and low access to anthropogenic food waste.

**Figure 3:**
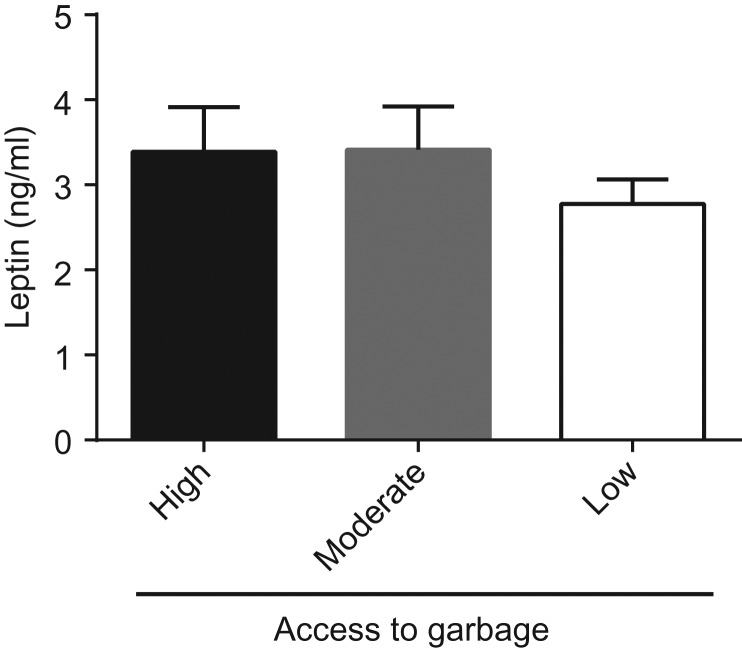
Effect of access to garbage on raccoon leptin concentration. Serum samples were assayed for leptin from raccoons with high, moderate and low access to garbage. Data is presented mean ± SEM in ng/ml. Data was analyzed using a one-way ANOVA. *n* = 23, *n* = 20 and *n* = 16 for the high, moderate and low access to garbage, respectively.

No significant difference was observed in serum leptin for the three groups of raccoons (*P* = 0.57, *F* = 0.56, df = 2, 52). A non-significant decrease was observed in the low access to anthropogenic food waste group compared to the moderate and high groups (Fig. 3).

We examined both body weight and GSP for differences between males and females. When comparing sex differences for raccoon body weight, a small but significant difference was found (*P* = 0.043, *F* = 4.31, df = 1, 54), however, there was no interaction between sex and the effect of access to anthropogenic food waste on body weight (*P* = 0.180, *F* = 1.77, df = 2,54). When comparing sex differences in the raccoon’s GSP concentrations, no effect was detected (*P* = 0.428, *F* = 0.64, df = 1, 53) and there was no interaction with access to anthropogenic food waste on GSP concentration (*P* = 0.644, *F* = 0.44, df = 2, 53).

**Figure 4:**
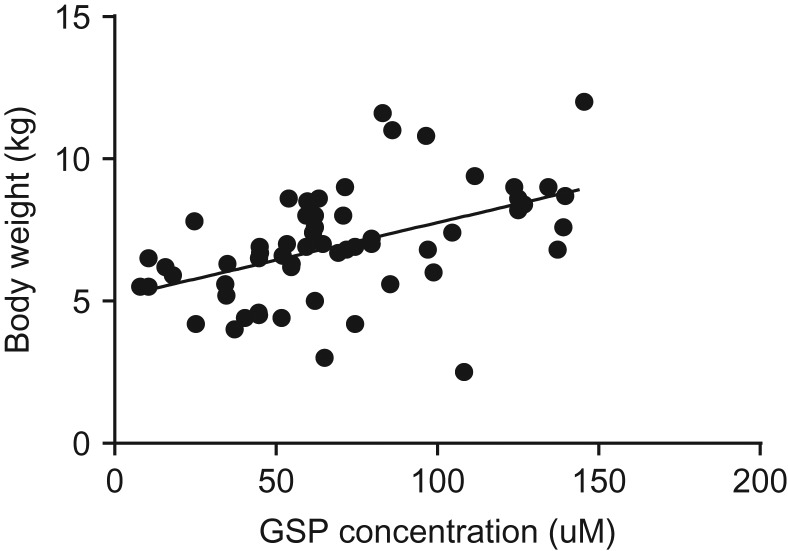
Relationship between raccoon body weight and GSP concentration. Adult raccoon (both male and female) GSP and body weight data were compared using Pearson’s correlation. *n* = 59, *r* = 0.4860, *P* < 0.001.

There was significant positive correlation between body mass and GSP (*n* = 59, *r* = 0.486, *P* < 0.001) (Fig. 4) but no such correlation existed between body mass and leptin (*n* = 59, *r* = 0.072, *P* = 0.60).

## Discussion

The consequences of human activities and the landscapes they modify for the nutritional physiology of wildlife are not well understood (reviewed in Birnie-Gauvin *et al.*, 2017), and there is little examination of the particular effects of a diet that is composed predominately of anthropogenic food waste. While there are several studies documenting the consumption of food waste in wildlife, and the ecological, life-history and population consequences of this consumption, there is very little known regarding how this novel diet affects the endocrine and metabolic functions of these species. Our investigation of urban and rural raccoons provides preliminary evidence that access to anthropogenic food waste is associated with a physiological response to this food resource. As we predicted, raccoons at the site with the highest availability of anthropogenic food waste were heavier and had about double the level of GSP than their counterparts with lower availability of food waste. Overall, our results indicate differences in glucose metabolism among sites that may be related to access to anthropogenic food waste.

Given previous work indicating an effect of access to anthropogenic food waste and body mass on leptin levels (Banks *et al.*, 2003), we expected our results related to GSP to be reflected in serum leptin. We further expected this because of previous work that showed a relationship between body mass on serum leptin in raccoons in Japan (Shibata *et al.*, 2005). Despite these expectations, serum leptin was not significantly different among our sites, nor was there an effect of body mass on serum leptin. Leptin is a marker for adiposity—that is, individuals with greater fat deposition are expected to have higher levels of serum leptin. There are several possible explanations for inconsistency between our predictions and results. First, variation in body mass of raccoons may not be related to variation in fat mass, but in other components, especially muscle mass. Second, the assays used in our study and Shibata *et al.* (2005) are different, and there may be differences in the reactivity of the antibodies used. Finally, our study is limited to raccoons during a single season, and of comparatively limited variation in body mass. Shibata *et al.* (2005) sampled raccoons throughout the year and the range in body mass of their sample of raccoons was larger than ours. Leptin has been investigated in relation to adiposity in other wildlife species, with conflicting results. Leptin is correlated with adiposity in black bears (*Ursus americanus*) (Spady *et al.*, 2009), but not white-tailed deer (*Odocoilieus viriginianus*) (Chitwood *et al.*, 2014). Further work should be done to determine the general utility of leptin as a marker for adiposity in wildlife.

Ultimately, the evolutionary consequences of changes in diet related to urban habitats must be investigated. Are there fitness consequences associated with hyperglycemia? Hyperglycemia itself may cause a reduction in reproduction and survival (although evidence of this is lacking in wild populations) or hyperglycemia may be indirectly linked to reductions in fitness. While the distribution of anthropogenic food resources, and the concomitant increase in population density, may facilitate disease transmission (Becker *et al.*, 2015; Flint *et al.*, 2016), chronic hyperglycemia itself may compromise immune function (Peleg *et al.*, 2007) and is associated with other chronic diseases (Barclay *et al.* 2008).

There is some evidence that urban populations are adapting to anthropogenic food sources. For example, recent work on urban populations of white-footed mice (*Peromyscus leucopus*) found genomic signatures of adaptation associated with the metabolism of lipids and carbohydrates (Harris and Munshi-South, 2017). These genomic patterns may be the result of adaptations for novel diets associated with cities—that is, anthropogenic food waste (Harris and Munshi-South, 2017). Similar results might be expected in urban raccoons that are reliant on similar food sources. For example, are the metabolic pathways associated with glucose metabolism or the metabolism of other nutrients such as lipids and carbohydrates affected by the urban diets? Are there differences in the frequency of alleles of candidate genes as demonstrated by Harris and Munshi-South (2017) in urban white-footed mice (*P. leucopus*)?

Our results suggest that access to anthropogenic food waste may affect glucose metabolism in urban raccoons. There is a number of research directions stimulated by this conclusion. First, a better measure of adiposity than body mass may be some measure of body condition (i.e. size-corrected mass (see Schulte-Hostedde *et al.*, 2001; 2005)) to control for differences in structural size. In addition, it would be interesting to know if there are differences in the patterns of adiposity (e.g. visceral vs. subcutaneous fat). We assumed that variation in body mass in our sample of raccoons would be the result of differences in fat mass, but there may be differences in body size or body composition instead. Perhaps most importantly, our estimates of consumption of anthropogenic food waste were qualitative and based on potential access to such food sources. More precise estimates of food consumption are required, and the use of stable isotopes to, for example, estimate corn consumption, would help determine the relative consumption of processed foods (e.g. Murray *et al.*, 2015). An expanded panel of biomarkers of metabolism including insulin, circulating lipids and pro-inflammatory cytokines would provide a more holistic perspective on the consequences of a novel, urban diet. Finally, a causative link between consumption of anthropogenic food waste and metabolic markers of metabolism will require a replicated study with quantified access to anthropogenic food waste.
